# AI-Based Detection of Coronary Artery Occlusion Using Acoustic Biomarkers Before and After Stent Placement

**DOI:** 10.1109/OJEMB.2025.3615394

**Published:** 2025-09-29

**Authors:** David Anderson Lloyd, Andrei Dragomir, Bulent Ozpolat, Biykem Bozkurt, Yasemin Akay, Metin Akay

**Affiliations:** Department of Biomedical EngineeringUniversity of Houston 14743 Houston TX 77004 USA; Department of NanomedicineHouston Methodist Research Institute 167626 Houston TX 77030 USA; Department of Medicine (Cardiology)Baylor College of Medicine 3989 Houston TX 77030 USA

**Keywords:** Artificial intelligence, coronary artery disease, acoustic signal processing, deep learning, matching pursuit

## Abstract

*Goal:* Cardiovascular disease is the leading cause of death in the USA. Coronary Artery Disease (CAD) in particular is responsible for over 40% of cardiovascular disease deaths. Early detection and treatment are critical in the reduction of deaths associated with CAD. *Methods:* Sound signatures of CAD vary for individual patients depending on where and how severe the blockage is. We propose the use of the artificial intelligence (AI, specifically the DeepSets architecture) to learn patient-specific acoustic biomarkers which distinguish heart sounds before and after percutaneous coronary intervention (PCI) in 12 human patients. Initially, Matching Pursuit was used to decompose the sound recordings into more granular representations called ‘atoms’. Then we used AI to classify whether a group of atoms from a single segment are from before or after PCI. Leveraging the model's learned latent representation, we can then identify groups of atoms which represent CAD-associated sounds within the original recording. *Results:* Our deep learning approach achieves a test-set classification accuracy of 88.06% using sounds from the full cardiac cycle. The same deep learning architecture achieves 71.43% accuracy using the isolated diastolic window sound segment alone. *Conclusions:* This preliminary study shows that individualized clusters of atoms represent distinct parts of heart sounds associated with occlusions, and that these clusters differentially change their spectral energy signature after PCI. We believe that using this approach with recordings from individual patients over many time points during disease and treatment progression will allow for a precise, non-invasive monitoring of an individual patient's condition based on unique heart sound characteristics learned using AI.

## I. Introduction

Cardiovascular disease (CVD) was the underlying cause of death for nearly one million people in the United States in 2021, and over 40% of those deaths are related to coronary artery disease [1]. Coronary artery disease (CAD) is a condition where the arteries which supply blood to the heart become obstructed by atherosclerotic occlusions [1], [2]. Cardiovascular disease is an enormous source of mortality, and it disproportionately affects Black Americans, 59% of Black adults in the United States having CVD [1]. In order to reduce the mortality of this disease and its impact on both patient lives and the healthcare system, early detection and treatment of CVD and CAD are critical.

Initial studies into acoustic detection of CAD showed that stenosis of the coronary arteries may give rise to diastolic murmurs [3], [4], [5]. Typically, catheterization or angioplasty were used to confirm the presence of stenosis at the site of the murmur, and the murmur was altered by the surgical intervention [6], [7]. It has been shown that coronary artery (CA) lesions can be detected reliably in diastolic window audio recordings [8], [9]. By isolating the diastolic window's sounds, our group was previously able to distinguish heart sounds as pre- or post-angioplasty with 91% accuracy in a cohort of 23 patients [10], [11]. Later, both fast Fourier transform (FFT) and autoregressive methods were shown to be useful for analysis of CAD acoustics using electronic stethoscopes [12], [13], [14].

We have previously investigated the detection of coronary artery occlusion via changes in heart sounds using electronic stethoscopes [15]. Using commercially available stethoscopes,we showed that both approximate entropy and the ratio between the power within the diastolic window of the fast Fourier transform (FFT) components above 150 Hz to those below 150 Hz, could reliably be used to identify if a patient was pre- or post-percutaneous coronary intervention (PCI). Patient treatment class was identified with 78% accuracy using this method [15], [16]. We then developed a novel microelectromechanical systems (MEMS) microphone array platform to record more detailed heart sounds of patients with a single coronary artery occlusion before and after PCI [16]. This array system recorded four separate channels of audio data which were then combined using delay-and-sum beamforming into a single low-noise recording. We calculated approximate entropy of the diastolic windows for eight human patients with single coronary artery blockages and two non-occluded patients. High approximate entropy values were associated with coronary artery disease ($p < 0.01$), and significant differences were found in the energy content of heart sound frequency components above and below 150 Hz ($p < 0.05$) [15], [16]. Furthermore, recent studies confirmed that previous approaches are useful for the detection of CA occlusions from audio containing occlusion-related turbulent flow [14]. We were also able to detect occlusion in not only the left coronary arteries that supply the anterior and lateral portions of the left ventricle, but also the right coronary artery that supplies the posterior part of the left ventricle and most of the right ventricle. These studies laid the groundwork for the audio detection of CAD through identification of cardiac-cycle acoustic biomarkers. However, these biomarkers of CAD will vary for each patient based on the severity and location of the blockage.

AI and ML have been employed to discriminate between sounds from hearts with or without CA occlusions, with recent approaches bridging the gap between simply achieving high classification accuracy and learning the actual acoustic features which represent the turbulence introduced by CA blockages [17], [18]. Interpretable AI approaches, including those using SHAP (SHapley Additive exPlanations), have been successful at the classification task using various transformations of the heart sounds features, such as spectrograms or Mel frequency cepstral components (MFCC) [18], [19], [20]. However, there is untapped potential in using this classification task to elucidate underlying task-related structure within the heart signals. Consequently, in this work we developed a reliable and explainable AI approach which can be implemented at a clinical level with a patient's physician to enable routine screening of CAD and the longitudinal monitoring individual patients' cardiovascular health.

Here we propose a novel approach leveraging the DeepSets architecture [21] to learn a patient-specific latent representation of heart sounds composed of Gabor Atoms from a matching pursuit (MP) [9], [22], [23] decomposition of beamformed heart sounds recordings [16]. We selected the DeepSets algorithm to facilitate the development of a permutation-invariant latent representation of Gabor atom heart sound decompositions. The resultant latent space shows excellent separability relative to the classification task, allowing for groups of Gabor Atoms to form semantic clusters whose power and presence within the signal change before and after treatment. While we were previously able to show aggregate changes in the acoustic signature for these patients before and after treatment, this method will allow us to characterize the specific acoustic signal changes in a patient's heart sounds after PCI. This technique can not only provide for advanced detection of CAD, but can be used to learn patient-specific signatures relating to their individual heart and disease state.

## II. Materials and Methods

### A. Patient Inclusion Criteria

Our group recruited subjects from the DeBakey VA Medical Center hospital according to and under the supervision of the Baylor College of Medicine IRB and the DeBakey VA Hospital R&D Committee as detailed in [16] and [15]. 12 subjects with CAD who were to undergo PCI for CAD at the DeBakey VA Hospital were enrolled. Patients were between 60 and 85 years of age, gave informed consent, had a body mass index (BMI) of less than 30, and had single-lesion CAD. Patients with heart valve disease, multi-vessel CAD, or implanted pacemakers/defibrillators were excluded. All patients enrolled in the study were male, reflecting to skewed patient population at the VA hospital.

### B. Heart Sound Recordings From Patients

As per our previous work, a recording platform was developed by our group with an array of four MEMS (micro-electromechanical systems) microphones to record the heart sounds of the patients. The development of this device is described in [16]. The device was placed six to eight cm dextral to the sternum midline, at the fourth intercostal space [16]. Patients were recorded for 15 seconds both before and after PCI. Patients were in a supine position during recording, and were instructed to hold their breath for the duration of the recording period. The device was placed at fourth intercostal and not moved or manipulated during recording. Signals were processed into two different datasets for a comparative deep learning approach (detailed in Section II-E).

### C. Raw Data Processing of the Heart Sounds

For each recording, the four channels were beamformed into a single 15 s signal according to the method described by our group in [16]. The final sound files were sampled at 4000 Hz. Each recording was then windowed into 1.5-second segments with 50% overlap for MP decomposition. MP was chosen because it represents these signals well and previously proved useful in the analysis of this data [16]. All portions of the signal were included, and windows were not aligned or shifted. Incidences of the second heart sound (S2) were identified for each patient using Springer's logistic regression hidden semi-Markov model (HSMM) method [24] trained on the PhysioNet dataset [25]. The diastolic window (DW) was defined as the first 128 milliseconds of audio data beginning 100 milliseconds after the onset of each S2, then decomposed into 100 atoms per DW segment.

### D. Matching Pursuit Decomposition

A software package was implemented in Python (v3.12.5) to construct a Gabor dictionary [23] using an FFT approach and use it to decompose a given signal into atoms. The full mathematical description of this process is included in the Supplemental Items. Each window was decomposed into a total of 100 atoms, or one sample.

### E. Feature Selection and Scaling

Each sample had nine corresponding features detailed in Table I. To account for non-centered time variance, an “atomic influence” feature is calculated for each atom as described in the Supplemental Items.

**TABLE I:** The Features for Each Atom Provided to the Machine Learning Algorithms. Coefficient Phase is Split Into a Sine and Cosine Component to Account for Circularity. Scalers Listed From Scikit-Learn (Version 1.5.2).

Atom Features			
Feature	Scaling Method	Unscaled Range	Scaled Range
Coefficient Magnitude	Standard Scaler	[0.12, 16.2]	[-0.9, 14.9]
Coefficient Phase (rad)	Phase Decomposition	[-$\pi$, $\pi$]	[-1, 1]
Frequency (Hz)	MinMax Scaler	[0, 1000]	[0, 1]
Octave	MinMax Scaler	[2, 12]	[0, 1]
Influence Given	None	[0, 54]	N/A
Influence Received	None	[0, 24]	N/A
Low Octave Influence	None	[0, 42]	N/A
High Octave Influence	None	[0, 30]	N/A
Patient ID	Label Encoding	[1, 12]	N/A

The sample labels were encoded, and shuffled into training, testing, and validation datasets (0.7:0.15:0.15) and batches of 16.

### F. DeepSets Architecture and Training

We implemented a variant of the permutation-invariant DeepSets architecture [21] in PyTorch, composed of an atom encoder, a shared multi-layer perceptron (MLP), a pooling mechanism, and an MLP output head. Our implementation allowed for tunable pooling methods (sum, mean, max, or attention pooling) as well as regularization through dropout and batch or layer normalization. Optuna [26] (v3.1.0) was used to perform hyperparameter optimization studies to maximize validation set accuracy. Hyperparameter search ranges are detailed in Table II. This architecture was used to train and optimize models for both the Full Cycle Heart Sound dataset and the Isolated Diastolic Heart Sounds dataset.

**TABLE II:** Optuna Was Used to Optimize Validation Accuracy for All Models With the Following Parameters. All Parameters Used Optuna's Range Searching Methods, With Learning Rate and Rate Decay Suggested Along a Logarithmic Scale.

Hyperparameter Search Space		
**Hyperparameter**	**Type**	**Range**
Latent Dimension (latent_dim)	Integer	[4, 512]
Hidden Dimension (hidden_dim)	Integer	[4, 512]
Pooling Mechanism (pooling)	Categorical	[’mean', ‘max', ’attention', ’sum']
Dropout Rate (dropout_rate)	Float	[0.0, 0.5]
Regularization (regularization)	Categorical	[’None', ‘LayerNorm', ’BatchNorm']
Learning Rate (learning_rate)	Float (log)	[1e-4, 1e-2]
Weight Decay (weight_decay)	Float (log)	[1e-6, 0.0]

### G. Latent Space Dimensionality Reduction and Clustering

After the training and hyperparameter optimization were completed, the model with the highest test set accuracy was used to generate atom latent representations. This representation was the output of the atom encoder layer, which was collected for all samples in the full dataset. Uniform Manifold Approximation and Projection (UMAP) components were calculated for each atom both for the entire cohort and for individual patients. We used UMAP instead of Principal Component Analysis (PCA) due to the nonlinear nature of the data and the poor fit of PCA to its underlying dynamics. Density-Based Spatial Clustering of Applications with Noise (DBSCAN) was used to cluster similar atoms ($\epsilon$ of 0.5, minimum of five points for dense region formation). The varying densities, robustness to noise, and non-circular shapes of the natural groups in the UMAP of the latent space made DBSCAN an ideal choice compared to approaches like K-means.

### H. Reconstructed Signal Processing

Signals reconstructed from atoms were smoothed with a Gaussian filter ($\sigma$ =5) and filtered with a 5th order Butterworth band-pass filter (65 Hz to 400 Hz) [16].

## III. Results

### A. Classification Accuracy Using the Full Cycle Heart Sounds

DeepSets models were assigned randomly generated code names for legibility. The top five models by testing set accuracy are shown in Fig. 2, with the highest accuracy for the testing set (88.06%) and the full dataset (94.05%) belonging to Lumpy-Chirpy-Kagu (LCK). LCK showed an F1-score of 0.87 and 0.89 on the pre- and post-PCI samples. A Random Forest model using an 80%:20% training:testing set split on the same dataset achieved 75% accuracy with an F1 score of 0.75 for both classes. This further confirms the utility of deep learning (especially set-based methods like DeepSets) for this type of data. Hyperparameters for LCK can be found in the Supplemental Items. High model accuracy and patient-wise specificity shown in Fig. 2 across the full dataset indicate the identification of treatment-critical information learned by the DeepSets model which is encoded within the learned latent space.

**Fig. 1.**
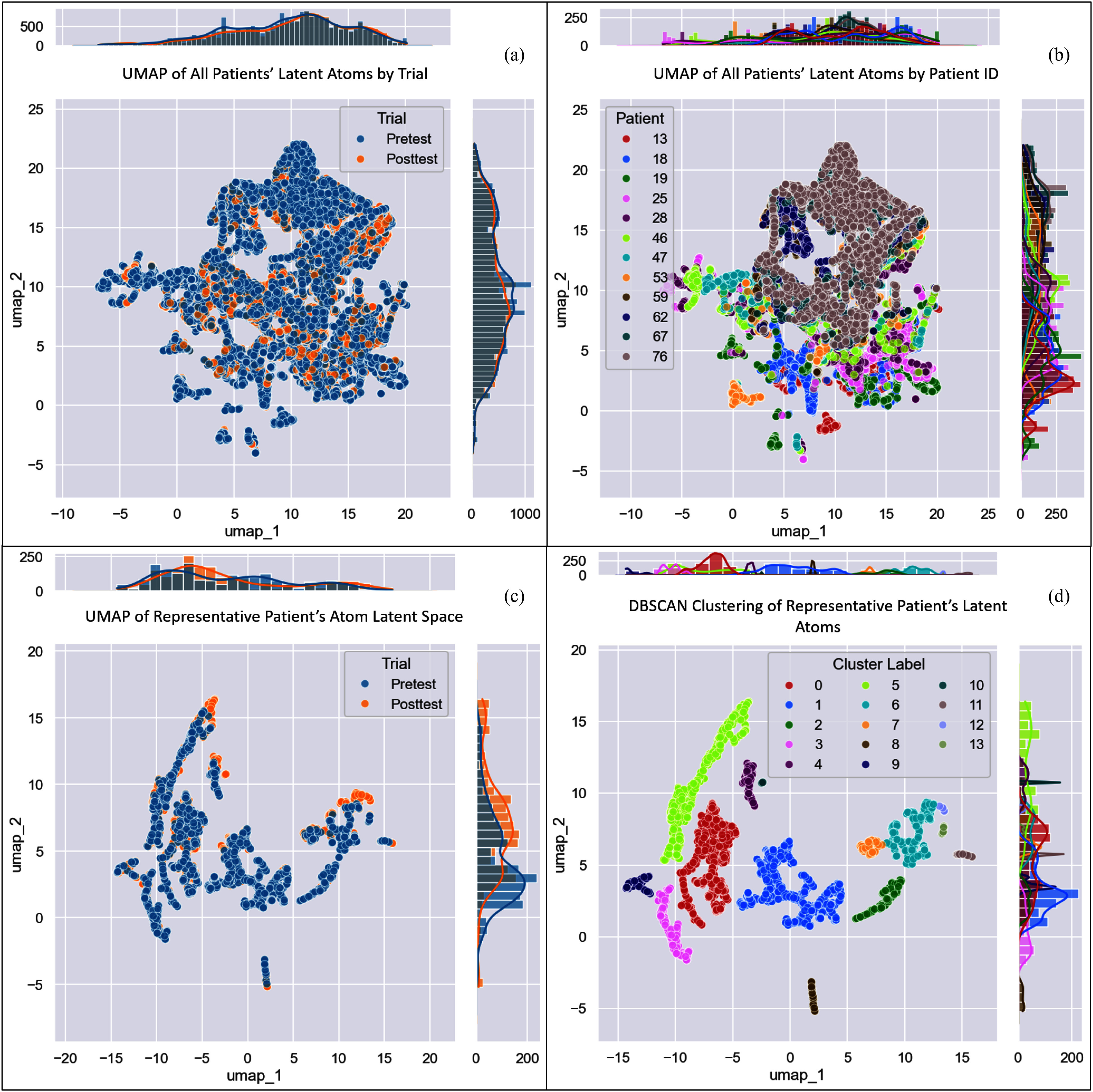
Atom Latent Representation UMAP. Full-dataset UMAP of all atom latent representations by (a) Trial Condition and (b) Patient ID. UMAP of all of the representative patient's atoms' latent representations by (c) Trial Condition and d) DBSCAN-clustered atoms (with an $\epsilon$ of 0.5 and minimum of 5 samples per cluster).

**Fig. 2.**
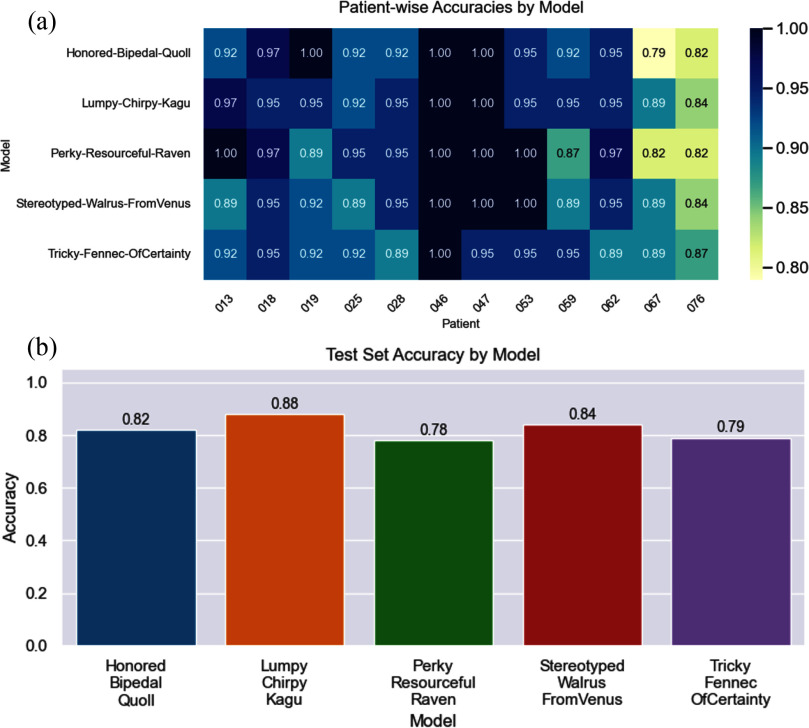
Patient-Wise Classification Accuracy using the Full Cycle of Heart Sounds. (a) The full dataset accuracy for each patient for the five models with the highest test set accuracy for the full-signal, sliding-window dataset. The model with the highest average accuracy and the highest test set accuracy was codename Lumpy-Chirpy-Kagu. (b) The test set accuracy for each model.

The latent space of LCK showed excellent stratification of patients (shown in Fig. 1(b)), reflecting a learned representation of patient acoustic characteristics. However, initial inspection of the latent space based purely on treatment status (shown in Fig. 1(a)) showed little readily visible separation in the UMAP.

By examining the atoms belonging to an individual patient (Patient 46), we can identify the patient-specific atom groups which correspond directly to changes in their individual cardiovascular acoustics. The histograms of the UMAP components shown in Fig. 1(c) illustrate the clear separation between treatment status on the second UMAP component axis. DBSCAN identified 14 clusters, labelled numerically from 0 to 13.

**Fig. 3.**
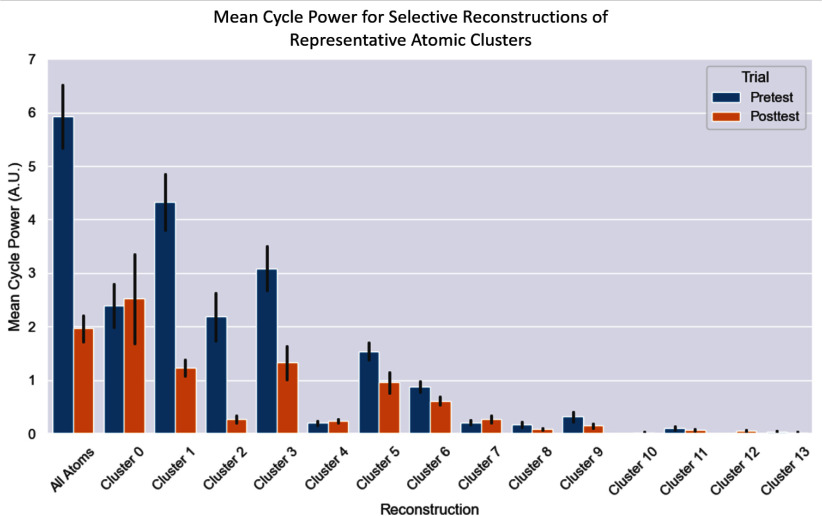
Change in Representative Patient Cluster Power before and after Treatment. The mean cardiac cycle power (diastole start to diastole start) for each cluster before and after PCI. Error bars depict standard error (SE).

**Fig. 4.**
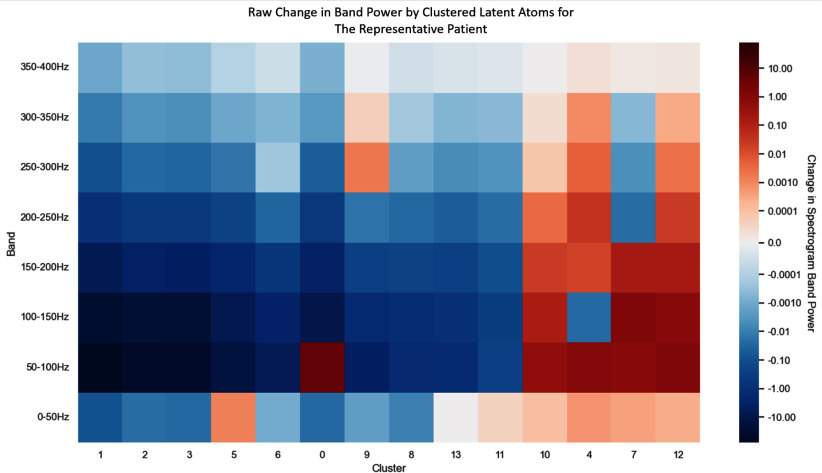
Representative Patient's Change in Cluster-wise Frequency Band Power. Mean change in short time Fourier transform spectral power within specific frequency bands before and after treatment. Clusters are sorted by ascending change in overall energy.

**Fig. 5.**
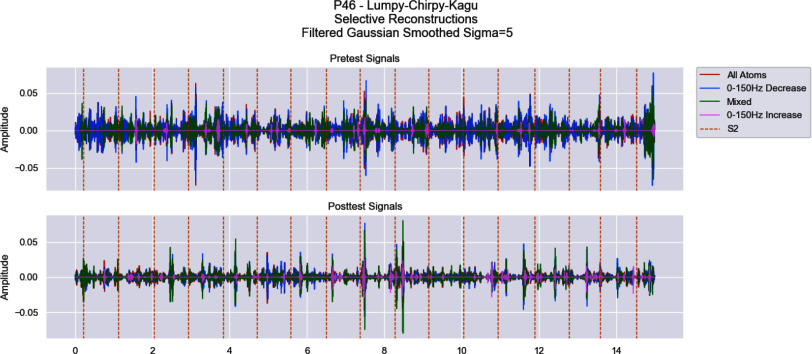
Representative Patient's Heart Sound Full Reconstruction. The top panel shows the cluster-specific reconstruction of the full 15 s recording both before (top) and after (bottom) PCI for the representative patient. Clusters are grouped based on whether their spectral power from 0–150 Hz monotonically increases or decreases within a $\pm 1e-6$ tolerance, while clusters with differential band responses are labelled “mixed”.

The signals were selectively reconstructed using atoms from individual clusters. Through the reconstructions, we can observe overall energy changes for each cluster after PCI (Fig. 3). Short time Fourier transform (STFT) was used to calculate the power in each frequency band for these reconstructions. Fig. 4 illustrates the raw change in energy for bands of 50 Hz (from 0 Hz to 400 Hz). There is a clear boundary between clusters which monotonically increase in power below 150 Hz, those which monotonically decrease in that range, and those which have a mixed response. Clusters were grouped based on monotonic change in energy within the 0–50 Hz, 50–100 Hz, and 100–150 Hz ranges and used to selectively reconstruct the full signal (shown in Fig. 5).

### B. DeepSets Training With Isolated Diastolic Heart Sounds

**Fig. 6.**
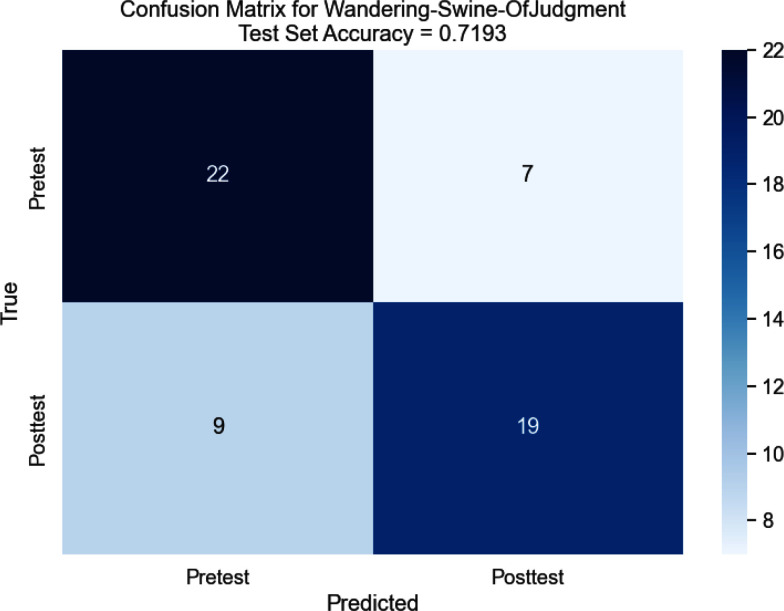
Best DW-Only Test Set Performance. Test set confusion matrix for the best performing DeepSets model trained on DW-only segments.

An equivalent Optuna optimization study (parameters in Table II) was performed on a model trained using the isolated DW segments rather than the full cycle (Section II-C). All of these showed poorer accuracy than the full-cycle sliding-window dataset, with the best model achieving a test-set accuracy of only 71.93% (F1 score of 0.73 for pretest and 0.71 for posttest) shown in the confusion matrix in Fig. 6.

## IV. Discussion

The primary goal of this work is differentiating task-relevant atoms from irrelevant ones. Pure atom count within a cluster is less meaningful for diagnostic interpretation than energy content (and change in energy content) is. In this work, decomposition was always performed to 100 atoms. Thus, when the occlusion is not present, atoms which previously represented it are freed to represent smaller acoustic details. Clusters 7, 10, and 12 are good examples of this. These clusters increase in energy in the representative patient's post-PCI recording since more atoms can be dedicated to the components they represent since the occlusion sounds are weaker. Clusters 1, 2, 3, 6, and 8 all monotonically decrease after implantation for all bands after implantation, implicating them in the representation of CAD-related acoustic biomarkers. The mixed and minimal change in clusters 5, 0, 13, 11, and 4 show their irrelevance to the occlusion. The fact that these biomarkers do not change substantially with implies that they represent sounds unrelated to CAD. These band-wise changes are illustrated in Fig. 4.

Likewise, the magnitude of an atom is not its primary semantic feature. Groups of atoms with various frequencies, octaves, *et cetera*, form a unit of meaning. Cluster 0, for instance, has a very small change in total energy. However, by examining its frequency makeup (See Fig. 4), we can see that the effect of treatment concentrates the cluster's energy from the full spectrum into the 50–100 Hz range. This study is predicated on the hypothesis that if a learned latent space is sufficiently separable and allows for accurate classification of samples, then that latent space must encode information on which atoms are most important for a model's decision. Furthermore, our results here show that this learned representation is patient-specific.

Using only the diastolic window, we previously achieved 78% accuracy [15], [16]. Using our DeepSets approach on the diastolic window segments we saw an accuracy of only 71.43%. However, by using the full cycle of heart sounds, the model had over 88% classification accuracy, despite its training set containing fewer samples than the DW-only one. These results imply that sound biomarkers indicating CAD are present throughout the entire cardiac cycle rather than in the diastolic window alone, and that including the full cycle sounds can improve classification accuracy.

The dataset used in this study had many patients, but only two recordings per individual. Adapting this method to a longitudinal study design (which follows a single patient's heart sounds over a longer period of time) allows for the evolution of the latent space over time to be characterized. If we hone in on the development of a latent space for a single patient across many sessions, we have the potential to both map drift in their precision biomarkers and detect new or worsening occlusions. The learned latent space continues to adapt to changes in patient physiology relating to both disease and treatment. We plan to explore the use of this platform to classify patients with multiple occlusions and use it to monitor the progression of these occlusions over longer time frames (such as multiple years). Furthermore, our MEMS microphone device is multi-channel by nature, which opens the possibility of learning channel-specific representations of occlusion, allowing us to focus on spatially specific biomarkers.

This preliminary study showed good performance in distinguishing sounds before and after PCI. We are currently working on the translation of this approach for use in the clinic to assess the recurrence of coronary occlusions after PCI. We are also interested in assessing and monitoring cardiovascular health of athletes and soldiers over time. We also believe that the latent space acoustic biomarkers could be useful to estimate the severity of occlusions. In a longitudinal clinical monitoring setting, the framing of the architecture will need to be adjusted to monitor changing dynamics. The focus would be to learn a model of an individual patient over multiple visits with their physician as both treatment and disease progress. Changes in these learned acoustic CAD biomarkers could then give quantitative metrics of patient status and progress. For example, if the CAD biomarkers decrease in strength over time, this indicates effective treatment. Likewise, strengthening of these signature implies worsening CAD. Even though the specific biomarkers for each patient are customized, the trends underlying improving or worsening patient condition are consistent through the population. These learned biomarkers would become a new source of information available to the clinician for more informed treatment design and management. This approach also has the potential to help detect and locate developing CAD in new patients, as detailed in the publications and approved/pending patents by our group and collaborators [15], [16].

## V. Conclusion

The novelty of our approach lies in the combination of Matching Pursuit decomposition, atomic influence features, and the clustering of atom groups within the learned atom encoding from DeepSets for the purpose of explicit time-domain-traceable interpretability of signal features. Clustering on dimensionally reduced data (especially through use of PCA) has been explored before [27], [28]. Deep learning models, particularly autoencoders, have proved effective in developing clustering-ready latent spaces [29], [30], [31], especially for image data. Set-based techniques incorporating attention components have been successful on multiple data modalities [21], [32]. Lee et al. extend the DeepSets architecture with attention components to develop their Set Transformer model [32]. They then perform clustering on the mixture of Gaussians of the set transformer's final output [32]. Our study focus on leveraging the knowledge generated during the learning process of a modified DeepSets model.

Matching pursuit has recently been combined with deep learning approaches for feature extraction and deep-learning-based representation development [33], [34], [35], but our use of matching pursuit to reversibly decompose a signal meaningfully grouped features is novel. We also show using the full cardiac cycle, rather than isolated segments, can substantially increase classification accuracy.

The ultimate goal of our group's investigations into CAD through cardiovascular acoustics has been to develop a noninvasive, passive, quick, and inexpensive way to identify and quantify CAD for individual patients. Ideally, such a method would be performed as a routine screening at regular check- ups. Our AI approach can be implemented at the clinical data level and allows for personalized, explainable, precision insights into a patient's cardiovascular health from just 15 seconds of heart sound data. This paradigm of empowering physicians and patients to understand their health and healthcare needs with AI assistance represents the next step in this goal, and in reducing the immense burden which cardiovascular disease has on health and quality of life.

## Supplementary Materials

Supplementary Materials

## Ethics Statement

All study and recruitment procedures were performed at the DeBakey VA Medical Center hospital according to and under the supervision of the Baylor College of Medicine IRB and the DeBakey VA Hospital R&D Committee. All patients provided informed consent to participate in the study.

## Conflicts of Interest

The authors declare no competing financial and/or non-financial interests.

## Author Contributions

MA, YA, BB, BO, AD, and DL, designed the experiments. DL performed the computational analysis. DL, YA, BO, BB, AD, and MA interpreted the data and wrote the manuscript. All authors have read and agreed to the published version of the manuscript.
