# Supplementary material for: AI-Based Detection of Coronary Artery Occlusion Using Acoustic Biomarkers Before and After Stent Placement
Source: IEEE Open J Eng Med Biol. 2025 Sep 29;6:557–63. doi: 10.1109/OJEMB.2025.3615394 (PMC12599900; doi:10.1109/OJEMB.2025.3615394)
Supplement: Supplementary Materials [file supp1-3615394.pdf]

# Supplementary Materials

## AI-Based Detection of Coronary Artery Occlusion Using Acoustic Biomarkers Before and After Stent Placement

David Anderson Lloyd, *Member, IEEE,* Andrei Dragomir, Bulent Ozpolat, Biykem Bozkurt, Yasemin Akay, *Senior Member, IEEE,* Metin Akay*, *LIFE Fellow, IEEE*

### I. Materials and Methods

#### A. Gabor Dictionary Construction

A software package was implemented in Python (v3.12.5) to construct a Gabor dictionary for a specified signal length according to the equations described in [1] using an FFT approach. The first step is to define Gaussian windows and then shift and scale them in frequency and time to create a multiscale Gabor dictionary. Window sizes were defined as

$$w_j = 2^j \quad \text{for } j \in \{2, 3, \ldots, \lfloor \log_2(N) \rfloor\}, \tag{1}$$

where $N$ is the length of the signal in points, and $j$ is the octave. The window sizes form a discrete set of powers of two, with $j$ taking whole-number values from 2 to $\lfloor \log_2(N) \rfloor$, ensuring that no window size exceeds the signal length. Each window's standard deviation $\sigma_j$ is expressed as

$$\sigma_j = \frac{w_j}{2\sqrt{2ln(2)}}. \tag{2}$$

Defining discrete time samples as $n$, the normalized deviation from the window center is

$$z[n] = \frac{n - \frac{w_j}{2}}{\sigma_j} \quad \text{for} \quad n \in \{0, 1 \ldots N-1\}, \tag{3}$$

and the Gabor windows for each octave are defined as

$$g_j[n] = e^{-\frac{1}{2} z[n]^2}, \tag{4}$$

where $g_j[n]$ is a centered Gabor window for a given octave. The windows are then scaled to have unit norm. Next we define the discrete time shifts and frequency shifts to convert the Gabor windows into atoms for our dictionary. Our Python implementation accepts a hop denominator parameter ($h_d$, default value of 2) to define the temporal granularity, so the resulting lists of time shifts for each octave $j$ is

$$u_j[k] = k \cdot \frac{w_j}{h_d} \quad \text{for} \quad k \in \{0, 1, \ldots, \lfloor h_d \cdot \frac{N - w_j}{w_j} \rfloor\}, \tag{5}$$

We calculate the frequency shifts using fast Fourier transform (FFT), defining our frequency bins as

$$\xi_j[m] = \frac{m \cdot f_s}{2w_j} \quad \text{for} \quad m \in \{0, 1, \ldots, w_j - 1\}, \tag{6}$$

where $f_s$ is the sampling rate of the signal (4000 Hz for these data). We use $2w_j$ to set the number of FFTs for the bins to account for zero padding in the operation. Finally, for each window $g_j[n]$ we shift and scale it by each time shift $u_j[k]$ and frequency shift $\xi_j[m]$, defining our Gabor atoms for each octave and shift index as

$$\phi_j[n, k, m] = g_j[n - u_j[k]] \cdot e^{2i\pi\xi[m]}, \tag{7}$$

applicable for all values of $n$, $k$, and $m$ defined in Equations (3), (5), and (6). Atoms are rescaled to unit norm. The combined set of all of these shifted and scaled Gabor atoms across all possible octaves gives us our multiscale Gabor dictionary as

$$D = \bigcup_j \{\phi_j[n, k, m]\}, \tag{8}$$

where the ranges of $n$, $k$, and $m$ are as defined in Equations (3), (5), and (6) for all $j$ defined in Equation (1).

#### B. Matching Pursuit Decomposition and Reconstruction

Each windowed signal was decomposed into 100 atoms, capturing an average of $97.881\% \pm 0.535$ of its energy. The decomposition of a signal into dictionary atoms is performed iteratively, as described in [1], [2], by performing a projection (scalar product) of each atom in the dictionary onto the signal. The atom with the maximum coefficient magnitude is selected and its scaled contribution to the signal is subtracted, producing a residual. This process is repeated iteratively on the residual signal until either a desired number of iterations, signal residual norm, or percentage energy capture is achieved. The complex projection coefficient, frequency, time center, and octave of each atom are stored, with the set of atoms representing the decomposed signal. Mathematically, the decomposition of a signal $x[n]$ over $A$ iterations is as follows. At each iteration $a$, the atom $\phi_{j_a}[n, k_a, m_a]$ is selected from the dictionary $D$ by maximizing the absolute value of the projection. This is defined as

$$c_a = \arg\max_{\phi \in D} \left| \sum_n R^{(a-1)}[n] \phi[n] \right|, \tag{9}$$

where $R^{(a-1)}[n]$ is the residual signal at iteration $a-1$, and $\phi[n]$ is an atom in $D$. The residual is then updated as for each iteration as

$$R^{(a)}[n] = R^{(a-1)}[n] - c_a \phi_{j_a}[n, k_a, m_a]. \tag{10}$$

Thus, the signal is decomposed into a set of selected atoms and coefficients. The signal can be reconstructed from these atoms and coefficients as

$$x_{rA}[n] = \sum_{a=1}^{A} c_a \phi_{j_a}[n, k_a, m_a] + R^{(A)}[n], \tag{11}$$

where $x_{rA}$ represents the signal reconstruction with $A$ atoms. This method allows for partial or selective reconstructions of the signal using specific atoms while excluding others.

Reconstructions of the 15-second signals are achieved by sequentially concatenating the reconstructions of each window belonging to that signal using the overlap-add method.

### C. Feature Scaling and Atomic Influence

The phase of the atom's projection coefficient was decomposed into sine and cosine components, resulting in two features instead of one. Providing the model with the specific time center of the atom can cause the model to either overrely on the time order of the features or cause it to fail to fit the features correctly. However, time synchrony of atoms is of vital importance to their semantic relevance in the context of the signal. We implemented an influence feature, taking into account the window size and center of each atom to define an influence range of the atom as $\frac{w_j}{2}$ points before its time center and after its time center, bounded by the signal length. If an atom's time center is in the influence range of another atom, it receives influence from that atom, and that atom gives it influence. We count the number of atoms giving and receiving influence to and from each atom, as well as the number of low octave (octaves 2 to 6) and high octave (octaves 7 to 12) influences given.

### D. Computer Specifications

Models were trained using Adam optimizer with 1e-5 weight decay and cross entropy loss on the model output. Training was conducted using a single NVIDIA Quadro RTX 4000 with 8GB of GDDR6 memory and 2304 CUDA cores.

## II. Results

### A. Lumpy-Chirpy-Kagu Best Hyperparameters

Final parameters for Lumpy-ChirpyKagu were an input dimension of 9, a latent dimension of 128, a hidden dimension of 128, a mean pooling mechanism, 0.5 dropout rate, no batch or layer normalization, and a learning rate of 0.001, trained for 2048 epochs.

## References

[1] S. Mallat, *A Wavelet Tour of Signal Processing: The Sparse Way*, 3rd ed. Amsterdam Boston: Elsevier/Academic Press, 2009.

[2] S. Mallat and Zhifeng Zhang, "Matching pursuits with time-frequency dictionaries," *IEEE Transactions on Signal Processing*, vol. 41, no. 12, pp. 3397–3415, December 1993.
